# Retrospective Study on the Clinical Superiority of the Vacuum-Assisted Closure System with a Silicon-Based Dressing over the Conventional Tie-over Bolster Technique in Skin Graft Fixation

**DOI:** 10.3390/medicina55120781

**Published:** 2019-12-12

**Authors:** Ping-Ruey Chou, Sheng-Hua Wu, Meng-Chien Hsieh, Shu-Hung Huang

**Affiliations:** 1 School of Medicine, College of Medicine, Kaohsiung Medical University, Kaohsiung 807, Taiwan; u105025047@gap.kmu.edu.tw; 2 Department of Anesthesiology, School of Medicine, College of Medicine, Kaohsiung Medical University, Kaohsiung 807, Taiwan; elsawu2@gmail.com; 3 Department of Anesthesiology, Kaohsiung Medical University Hospital, Kaohsiung 807, Taiwan; 4 Department of Anesthesiology, Kaohsiung Municipal Ta-Tung Hospital, Kaohsiung 807, Taiwan; 5 Division of Plastic Surgery, Department of Surgery, Kaohsiung Medical University Hospital, Kaohsiung 807, Taiwan; williehsieh@hotmail.com; 6 Department of Surgery, School of Medicine, College of Medicine, Kaohsiung Medical University, Kaohsiung 807, Taiwan; 7 Regeneration Medicine and Cell Therapy Research Center, Kaohsiung Medical University, Kaohsiung 807, Taiwan

**Keywords:** VAC, silicon-based dressing, pain, skin graft

## Abstract

*Background and Objectives*: The tie-over bolster technique has been conventionally used for skin graft fixation; however, long operative times and postoperative pain are the main disadvantages of this method. In this study, we introduce a new method using vacuum-assisted closure (VAC) with a silicon-based dressing as an alternative for skin graft fixation. This retrospective study aimed to evaluate the clinical effect of the VAC plus silicon-based dressing method and the conventional tie-over bolster technique for skin graft fixation in terms of pain, operative time, and skin graft take rate. *Materials and Methods*: Sixty patients who underwent skin graft surgery performed by a single surgeon from January 2017 to October 2018 were included in this clinical study. They were divided into two groups based on the type of treatment: tie-over bolster technique and vacuum-assisted closure (VAC), or silicon-based dressing groups. The operative times were recorded twice (during suturing or stapling of the graft and during removal of the dressing) in the two groups; similarly, pain was assessed using a numeric rating scale (NRS) after surgery and during dressing removal. Skin graft take rate was evaluated two weeks after dressing removal. *Results*: Twenty-six patients who met the eligibility criteria were enrolled into the study and assigned to one of the two groups (n = 13 each). No significant differences in age, gender, and graft area were noted between the two groups of patients. The VAC plus silicon-based dressing group demonstrated higher skin graft take rates (*p* < 0.05), shorter operation times (*p* < 0.05), and lower levels of pain (postoperative pain and pain during dressing removal) compared with the tie-over bolster technique group (*p* < 0.05). *Conclusions*: These findings indicate that VAC with silicon-based dressing can be used for skin graft fixation due to its superior properties when compared with the conventional method, and can improve the quality of life of patients undergoing skin graft fixation.

## 1. Introduction 

Skin grafts are widely used for reconstructive surgery in patients with burns and traumatic or iatrogenic defects that cannot be closed [1]. Optimal fixation of skin grafts is necessary for a successful reconstruction [2]. The conventional tie-over bolster technique with splinting is one of the most commonly-used immobilization techniques for fixing the graft to the wound bed to achieve adequate postoperative graft adherence [3]. However, this technique has some disadvantages for both patients and clinicians. Patients may experience postoperative pain due to the tension of the bolster sutures and tearing pain during the removal of the tie-over dressing [4]. Postoperative pain is highly related to the tension caused by the silk or nylon sutures used in the tie-over bolster technique, and tearing pain is mainly due to the adhesion of the inner layer of the petrolatum gauze to the tissues [5]. Splints are used when the tie-over method is applied to the extremities. However, the heavy and uncomfortable feeling caused by these structures must be taken into consideration during postoperative wound care; furthermore, the splints may add to unnecessary medical waste [4]. For clinicians, placing sutures around the periphery to affix the graft to the wound bed is a time-consuming and challenging procedure [6]. Additionally, secondary trauma during removal due to the adhesion of the dressing to the wound bed can impair the graft outcome [7]. Vacuum-assisted closure (VAC), also known as negative pressure wound therapy, has been used as a dressing for patients with open-fractures and acute or chronic burn wounds to reduce the extent of the injury [8]. The postoperative use of VAC can create a controlled and closed environment and reduce exudation, thereby facilitating local circulation and tissue granulation [9]. For skin grafts, VAC can be applied as a bridge for grafting, as a fixation method to secure the graft, and as a dressing at the graft donor site [8]. Additionally, this technique has proven to save time for clinicians and improve patient mobility due to its favorable fixation ability and the absence of a splint [9]. Furthermore, patients with VAC reported less postoperative pain and less use of analgesics compared with those who used conventional dressings [10]. However, the issue of tearing pain during the removal of the dressing continues to pose a challenge.

For decades, the requirement for an ideal primary wound contact material with nonadherent and atraumatic properties that can provide an optimal environment between the wound bed and the dressing has been indicated; silicon-based wound dressings were reported to have the ability to address the issues of tissue adherence, pain, and trauma [7]. To overcome the clinical disadvantages of the tie-over and VAC techniques for skin graft fixation, the use of silicon-based dressings may prove useful for reducing pain during the removal of the dressing. This retrospective study aims to demonstrate that the use of VAC with a silicon-based dressing can not only save operative time, but can also reduce postoperative pain, as well as pain which occurs during dressing removal, compared with the conventional tie-over bolster technique for skin graft fixation.

## 2. Methods

### 2.1. Patient Selection 

**Figure 1:**
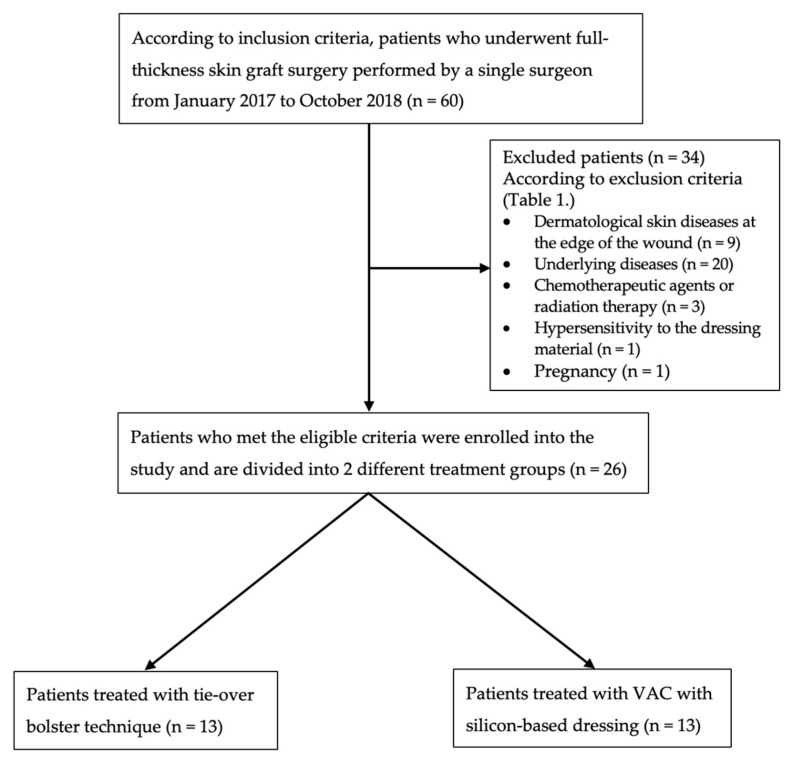
Flowchart of patient selection algorithm based on inclusion and exclusion criteria (Table 1.) and two treatment groups (tie-over bolster technique and VAC with silicon-based dressing).

Twenty-six out of a total of 60 patients who underwent skin graft surgery performed by a single surgeon from January 2017 to October 2018 were included and evaluated in this retrospective clinical study (Figure 1). The study was approved by the Institutional Review Board of Kaohsiung Medical University in Taiwan (KMUHIRB-E(I)-20190190; approval date 25 June 2019). The individuals in this manuscript have given written informed consent to publish these case details presented below.

The inclusion and exclusion criteria for the patients in this study are listed in Table 1. The demographic data of the patients, including age, gender, graft area, graft fixation method on the wound bed, the use of a splint, operative time, donor site, and pain assessment levels, are summarized in Table 2. 

### 2.2. Surgical Technique

The patients received skin grafts, which were fixed using either the tie-over bolster technique or the VAC with the silicon-based dressing for four days. 

#### 2.2.1. Tie-over Bolster Technique 

**Figure 2:**
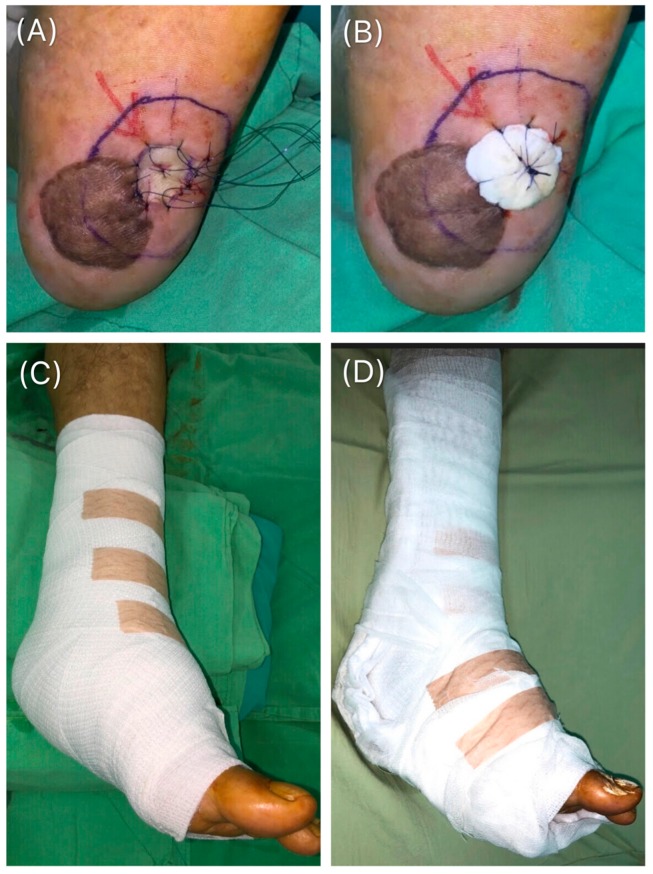
Tie-over bolster technique. (**A**) The graft was fixed on to the wound bed using 3-0 or 4-0 nylon and anchoring sutures placed at intervals of 4 to 10 mm. (**B**) A petrolatum gauze covered with cotton balls was tied over the sutures to prevent graft slippage. (**C**) and (**D**) A splint was applied to the extremities.

In this technique, the graft must be laid over the entire surface of the wound bed without stretching or wrinkling it. First, the graft was sutured using 3-0 or 4-0 nylon anchoring sutures, depending on the surface of the wound bed (Figure 2A). The distance between each suture was 4–10 mm. The operative time for graft fixation to the wound bed was recorded in seconds (s). Subsequently, petrolatum gauze covered with cotton balls were tied over the sutures with the aid of an assistant who held on to them to prevent slippage. The wound was covered with a cotton roll and bandage (Figure 2B). A splint was applied if the graft was located at the extremities and the time taken for its installation was recorded (Figure 2C,D). Four days after surgery, the tie-over was removed at the bedside and the site was examined. This technique had been widely used for graft fixation because it ensures contact between the graft and the wound bed, thereby preventing hematoma accumulation [11]. 

#### 2.2.2. VAC Therapy with Silicon-based Dressing 

**Figure 3:**
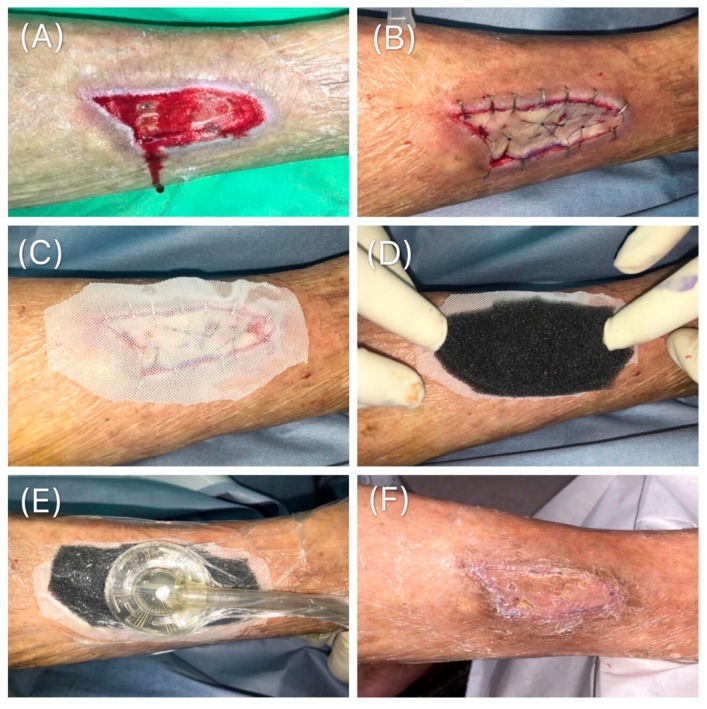
VAC with silicon-based dressing. (**A**) Wound requiring full-thickness skin graft. (**B**) After the graft was placed over the wound bed, staples were applied for graft fixation. (**C**) The graft was then covered directly with a silicon-based dressing. (**D**) A black sponge was trimmed to cover the silicon-based dressing and apply the VAC. (**E**) The VAC was applied with a negative pressure of 50 mmHg. (**F**) Four days after the operation, the VAC was removed.

After the skin graft was placed over the wound bed (Figure 3A), staples were used for graft fixation (Figure 3B). The time taken for the fixation was recorded in seconds. The graft was then covered directly with a silicon-based mesh (SI-mesh; ALCARE Tokyo, Japan; Figure 3C), followed by the placement of the VAC dressing (INFOV.A.C. ^™^ Therapy system, KCI, an Acelity Company, San Antonio, Texas) on top of it (Figure 3D). The operative time for the application of the dressing was recorded and negative pressure (50 mmHg, set in continuous mode) was applied (Figure 3E); this amount of pressure can be tolerated without compromising the outcome of the skin graft [12]. Four days after surgery, the VAC dressing was removed (Figure 3F). A supported operation video is presented in Video S1. 

#### 2.3. Operative Time

Operative times were recorded at the time of graft fixation (using sutures or staples) and at the end of the application of the VAC plus silicon-based dressing or the tie-over plus splint technique (Table 2). 

#### 2.4. Pain Assessment 

The numeric rating scale (NRS) is one of the most commonly-used scales for measuring pain in clinical settings. It uses Arabic numbers from 0 (no pain) to 10 (the worst imaginable pain) to quantify pain [13]. The NRS was used to assess postoperative pain when the patient returned from the operation theater and pain on dressing removal on day 4 when the dressing was removed.

#### 2.5. Skin Graft Take Rate 

The skin graft “take” rate was evaluated by the surgeon as ((total graft area) − (graft loss area))/((total graft area) × 100) two weeks after dressing removal. The graft loss area was determined by inspecting the areas where the graft had failed to adhere to the wound bed or where graft tissue necrosis had occurred.

#### 2.6. Statistical Analysis 

All data were recorded using Microsoft Excel for Mac, version 16.23 (190309), which was used for the statistical analyses in this study. The Kolmogorov-Smirnov test was applied to evaluate the normality of distribution of the investigated parameters. All parameters in our study were consistent with normal distribution. Data were expressed as mean ± standard deviation. Comparisons between patients using the tie-over and VAC methods were performed using two-tailed t-test. The value *p* < 0.05 was considered statistically significant. 

## 3. Results 

### 3.1. Patient Demographics

Sixty patients underwent skin graft surgery from January 2017 to October 2018. After the exclusion of patients based on the exclusion criteria, a total of 26 patients were included in the study. Wound etiology of our patients included various types of trauma on lower extremities which required full-thickness skin grafts, such as necrotizing fasciitis, surgical defects of skin cancer, burns, dog bites, and diabetic wounds. Before skin grafting, every wound bed received VAC therapy until well granulation formation. The tie-over bolster technique plus splint group comprised 13 patients (men, 9; women, 4) with a mean age of 50.7 ± 22.5 years and a mean graft area of 19.3 ± 9.3 cm^2^. In the VAC plus silicon-based dressing group, there were 13 patients (men, 10; women, 3) with a mean age of 48.8 ± 21.6 years and a mean graft area of 23.5 ± 8.3 cm^2^. No significant differences in age, gender, and graft area size were noted between the two groups of patients. The characteristics and demographics of the patients in the two groups are summarized in Table 2. 

### 3.2. Operative Time

**Figure 4:**
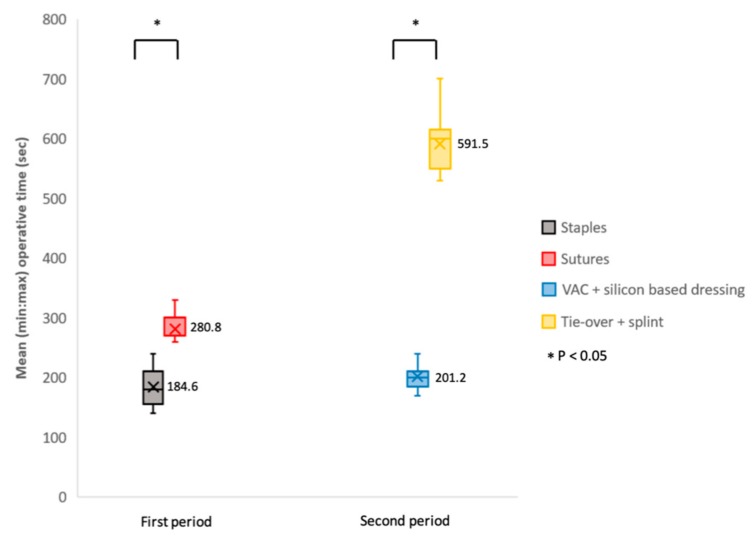
Operative time (s) comparison of graft fixation using staples or sutures (first period) and application of VAC plus silicon-based dressing or tie-over plus splint (second period). Data were presented in box and whisker plot (minimum, first quartile, median, third quartile, and maximum) and conducted by two-tailed t-test for comparison. Mean values are highlighted as a cross marked in every box with an accompanying Arabic numeral.

As shown in Table 2 and Figure 4, the mean operative time for graft fixation in the group that used sutures (tie-over bolster plus splint) was significantly (*p* < 0.05) longer at 280.8 ± 51.7 s than that in the group that used staples (VAC plus silicon dressing) at 184.6 ± 33.6 s. 

Similarly, the second mean operative time in the tie-over plus splint group was significantly (*p* < 0.05) longer (mean, 591.5 ± 162.9 s) than that in the VAC plus silicon dressing group (mean time, 201.2 ± 54.4 s).

### 3.3. Pain

**Figure 5:**
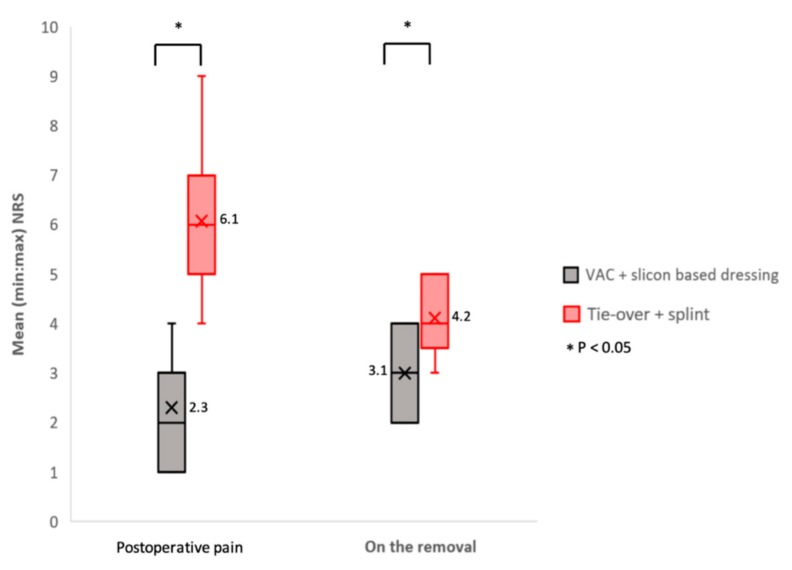
Pain assessment (NRS) comparison at two different time points: when the patient returned form the operation theater (postoperative pain) and on day four, when the dressing was removed (on the removal). Data were presented in box and whisker plot (minimum, first quartile, median, third quartile, and maximum) and conducted by two-tailed t-test for comparison. Mean values are highlighted as cross marked in every box with an accompanying Arabic number.

As illustrated in Table 2 and Figure 5, the mean NRS for postoperative pain was 6.1 ± 2.3 in the tie-over plus splint group and 2.3 ± 3.0 in the VAC plus silicon dressing group (*p* < 0.05). Similarly, the NRS for pain on dressing removal in the tie-over plus splint group was higher (mean, 3.1 ± 2.8) than in the VAC plus silicon dressing group (mean, 4.2 ± 2.4; *p* < 0.05). 

### 3.4. Skin Graft Take Rate

**Figure 6:**
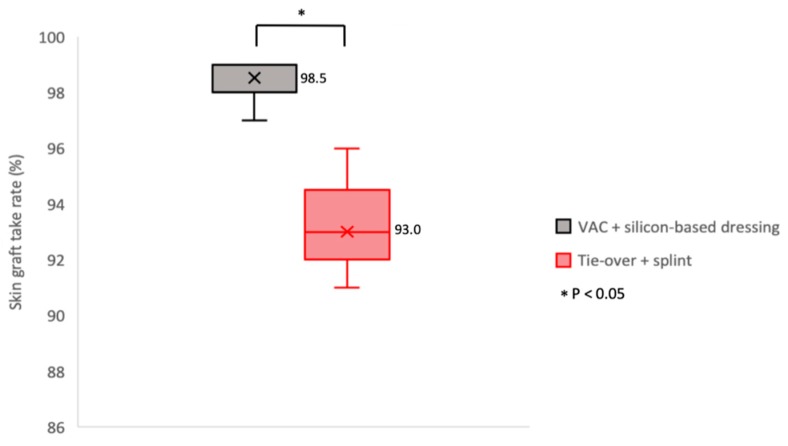
Skin graft take rate (%) comparison between the VAC plus silicon-based dressing and tie-over plus splint groups as two weeks after dressing removal. Data were presented in box and whisker plot (minimum, first quartile, median, third quartile, and maximum) and conducted by two-tailed t-test for comparison. Mean values are highlighted as cross marked in every box with an accompanying Arabic number.

As summarized in Table 2 and Figure 6, in the tie-over plus splint group, the mean skin graft take rate was 93.0% ± 21.4%, which was significantly (*p* < 0.05) lower than that in the VAC plus silicon-based dressing group (mean take rate, 98.5% ± 22.9%). 

## 4. Discussion

In the present study, we compared the effectiveness of the VAC plus silicon-based dressing with the conventional tie-over bolster technique in patients who underwent skin graft surgery. 

The tie-over bolster technique was introduced by Langtry et al. as an effective graft fixation method to reduce the incidence of postoperative wound hematoma and bleeding, and to enhance the adherence of the graft [14]. A recent study by Kromka et al. emphasized that this technique could be used to secure grafts to the wound bed, depending on the anatomic location and the preference of the surgeon performing the procedure [15]. It is challenging to perform a full-thickness skin graft (FTSG) on the lower extremities owing to the inevitable shearing forces associated with patient mobility, which can jeopardize the graft take rate and survival [3]. Struk et al. conducted a study to evaluate the outcome of using the tie-over technique with a noncircular plaster cast as a postoperative immobilization method for covering lower leg defects [3]. The use of a noncircular plaster cast or custom-made splint with the tie-over technique minimized shearing force in the patients in their study, thereby indicating that a shorter immobilization period may improve FTSG survival [3]. In the current study, a splint was used with the tie-over method for postoperative immobilization in 13 patients, but the additional weight of splint restricted the overall mobility of these patients. Studies have shown that the tie-over technique does not exert pressure in an even manner at the interface between the graft and the wound bed; therefore, its conventional benefits as a “pressure graft fixation dressing” are diminished [15]. VAC has been frequently discussed as an alternative method for such surgeries in many recent studies. 

Korber et al. conducted a study on the postoperative use of VAC on a mesh graft in patients with chronic ulcers and found that the healing rate was significantly higher in the VAC treatment group compared with the conventional dressing group [9]. Similarly, studies have also reported that a VAC dressing without a splint can effectively produce a uniform compressive force and firmly fix the graft in place compared with the conventional bolster dressing [16,17]. Waltzman et al. carried out a large sample study to reveal that split-thickness skin grafts (SFSGs) can be well-secured with VAC, especially in patients with burn injuries. Additionally, they demonstrated that VAC could be used in locations that were anatomically challenging for the placement of conventional bolster dressings, such as the trunk, thighs, and other contoured areas [18]. The SFSG take rates of VAC and conventional bolster dressings were reported to be 96% and 89%, respectively by Scherer et al. [19] and 97.9% and 79.2%, respectively by Nakamura et al. [20]. In another study, VAC was reported to improve the SFSG rate by improving the revascularization process and preventing the accumulation of seroma or exudate (which can interfere with adhesion) beneath the graft [8]. Shin et al. retrospectively reviewed 10 FTSG cases from the anterolateral thigh (ALT) with the aid of a postoperative VAC, and reported that the thick skin of the FTSG possessed more tension than that of the STSG, resulting in uneven pressure on the wound bed and subgraft fluid accumulation; thus, the use of VAC is feasible for improving FTSG outcomes [21]. The decrease in operative time using VAC compared with the tie-over bolster technique was demonstrated in a study by Nakamura et al. [20]. Nonetheless, the limitations of VAC include additional short-term medical costs and adhesion during dressing removal. The increase in short-term costs may be compensated for by the significant reduction in postoperative treatment duration, which can lead to a reduction in the overall long-term costs compared with conventional bolster dressing [9]. However, the adhesion of the sponge dressing of the VAC to the graft during dressing removal causes damage to the graft tissue and pain for the patient [21]. 

In a study conducted by Wang et al., surgical procedures that took longer than expected to complete resulted in increased rates of postoperative complications compared with procedures that were completed within a short period of time [22]. It is crucial to educate the operation theater staff, improve preoperative planning, and employ efficient surgical methods to decrease the effect of operative time on surgical outcomes [23]. Furthermore, a systematic review and meta-analysis by Cheng H et al. showed that a reduction in operative time can significantly reduce the overall costs of the surgical procedure [24]. Thus, both time- and cost-effectiveness are crucial for the clinician while making surgical decisions [20]. 

Longer operative time is one of the technical disadvantages of the conventional tie-over bolster technique due to the additional materials and application steps involved [4]. Akihiko et al. suggested the use of a polyurethane foam and elastic tapes to shorten the operative time, reduce medical waste, and produce likely graft outcomes, instead of using the conventional continuous sutures and knots on bolsters [25]. The superiority of VAC over the tie-over bolster technique (better skin graft survival and reduced operative time) was demonstrated in a recent study [20]. A similar finding was observed in the current study, wherein the installation time for VAC was shorter than that for the conventional tie-over bolster technique (*p* < 0.05) due to the straightforward application procedure for the VAC system. Moreover, VAC can be performed by a single surgeon without the need of an assistant.

According to related studies, postoperative wound pain can lead to psychological depression, which may result in physiological stress and delay wound healing [7,26]. The psychological results and somatic symptoms of depression that are not normally felt by the sensory route can be interpreted as disgraceful or even painful [27], resulting in a vicious cycle, where the clinical situation deteriorates before wound care is appropriately managed [26]. In the current study, the conventional tie-over bolster technique produced significantly higher levels of postoperative pain (*p* < 0.05) when compared with the VAC plus silicon-based dressing method. Based on clinical experience, we propose two explanations for this result: first, the tension from the nylon or silk sutures surrounding the perigraft area may have caused a feeling of contraction on the skin. Second, the splint used on top of the tie-over bolster may have added to the weight, making it bulky and uncomfortable for the patient. The patient might interpret both of these sensations as pain. 

Conventional wound dressings such as paraffin gauze pose as physical disturbances for the wound bed during dressing change [7]. The presence of the exudate might aid in the sticking of the gauze to the wound; subsequently, capillary loops and granulation tissue may grow through the fabric components of the dressing during the course of wound healing [7], resulting in a tearing pain and trauma to the fragile tissues during the removal of the dressing [28,29]. To date, recommendations for the prevention of pain and tissue disturbance have focused on the use of nonadherent dressings (e.g., silver-coated, silicon, hydrofibers, and polyacrylamide) [7]. These dressings can lower adherence without compromising antimicrobial activity and increasing cytotoxicity [30]. A silicon-based dressing is reported to be better than conventional paraffin gauze due to its nonadherence to the graft bed [31]. The prime advantage of the soft silicon wound contact layer is that it is pain-free and atraumatic for the graft and the wound bed during dressing removal [32]. Mepitel^®^, a lipidocolloid soft polymer and silicon-coated dressing, demonstrated no disturbance to the wound, and patients did not experience discomfort during dressing removal [33]. Moreover, this dressing could protect the treated skin from dermal irradiation [34]. Mepitel^®^ and a secondary dressing, which was applied with the aim of eliminating exudate infiltration to minimize eschar formation, was first applied to pediatric burn wounds [35]. 

In 2016, Robert et al. reviewed the indications of VAC and introduced the use of a silicon interface between the wound and a black foam of VAC to protect the fragile, exposed tissue and reduce pain on removal [36]. Compared with our study, reduced pain on removal is the main advantage of the additional use of a silicon-based dressing. Furthermore, pain assessment was conducted for comparison with the conventional tie-over bolster technique. In the present study, the silicon-based dressing with VAC produced significantly less tearing pain during dressing removal compared with the conventional tie-over bolster technique. This may be attributed to the fact that silicon-based dressings can promote compliance by decreasing overall trauma to the wound bed. Furthermore, the VAC system is easy to dismantle, depending on the skill and proficiency of clinician. Additionally, the VAC plus silicon-based dressing can ensure that the graft is in contact with the wound bed throughout the process. Thus, the survival of the graft is enhanced, resulting in an increase in the quality of postoperative wound care.

### Restrictions of the Study

This study has some limitations in terms of the wound type, the sample size, the lack of a cost analysis, and length of the follow-up period. Ideally, a larger population of participants with various types and sizes of wounds would provide more clinical integrity. Cost analysis, which includes medication and extra nursing time spent on postoperative pain management, should be taken into consideration in future studies. Furthermore, a longer follow-up time is recommended to determine patients’ quality of life and to quantitatively assess their mobility. We aim to conduct further research to investigate the application of VAC plus silicon-based dressings in various clinical scenarios.

## 5. Conclusions

The results of this retrospective study revealed that the VAC system with silicon-based dressing causes less postoperative pain, is less time-consuming, and has better skin graft take rates than the conventional tie-over bolster method. Thus, it exerts clinical superiority over the conventional technique as an effective skin graft fixation method for postoperative wound care management.

## Figures and Tables

**Table 1:** Inclusion and exclusion criteria.

**Inclusion criteria**
Patients presenting with a wound that requires a full-thickness skin graft Graft fixation with VAC + silicon-based dressing or tie-over bolster technique Participants aged 18 to 80 years Participants with graft area of 5 cm^2^ to 100 cm^2^ Operation performed by a single surgeon
**Exclusion criteria**
Participants with dermatological skin diseases at the edge of the wound Participants with underlying diseases such as SLE, hepatic failure, HIV/AIDS, malignant neoplasm, or severe anemia Patients using immunosuppressive or chemotherapeutic agents, or those undergoing radiation therapy Participants who were allergic to or had a hypersensitivity to the dressing material Participants who were pregnant

VAC, vacuum-assisted closure; SLE, systemic lupus erythematosus; HIV, human immunodeficiency virus; AIDS, acquired immunodeficiency syndrome.

**Table 2:** Patient Demographics of skin graft under VAC with silicon-based dressing or tie-over bolster technique.

Variables	VAC with Silicon-based Dressing (n = 13)	Tie-Over Bolster Technique (n = 13)	*p*
**Age (yrs)**			
Mean	48.8	50.7	0.8
SD	21.6	22.5	
**Gender (cts)**			0.67
Male	10	9	
Female	3	4	
**Graft area (cm^2^)**			
Mean	23.5	19.3	0.23
SD	8.3	9.3	
**Graft fixation on wound bed (case cts)**			
Staple	13	0	
Suture	0	13	
Splint using	0	13	
**First operative time (secs)**			
Mean	184.6	280.8	< 0.05*
SD	33.6	51.7	
**Second operative time (secs)**			
Mean	201.2	591.5	< 0.05*
SD	54.4	162.9	
**Postoperative pain (NRS)**			
Mean	2.3	6.1	< 0.05*
SD	3.0	2.3	
**Pain on the removal (NRS)**			
Mean	3.1	4.2	< 0.05*
SD	2.8	2.4	
**Skin graft take rate (%)**			
Mean	98.5	93.0	< 0.05*
SD	22.9	21.4	

VAC, vacuum-assisted closure; yrs, years; cts, counts; secs, seconds; SD, standard deviation; NRS, numeric rating scale; ds, days; %, percentage; *, significant difference (*p* < 0.05, conducted by two-tailed t-test).

## Supplementary Materials

The following are available online at https://www.mdpi.com/1010-660X/55/12/781/s1, Video S1: Operation video.
